# Quality control procedures and metrics for resting-state functional MRI

**DOI:** 10.3389/fnimg.2023.1072927

**Published:** 2023-03-13

**Authors:** Rasmus M. Birn

**Affiliations:** ^1^Department of Psychiatry, University of Wisconsin-Madison, Madison, WI, United States; ^2^Department of Medical Physics, University of Wisconsin-Madison, Madison, WI, United States

**Keywords:** connectivity, motion, fMRI, artifacts, quality control

## Abstract

The monitoring and assessment of data quality is an essential step in the acquisition and analysis of functional MRI (fMRI) data. Ideally data quality monitoring is performed while the data are being acquired and the subject is still in the MRI scanner so that any errors can be caught early and addressed. It is also important to perform data quality assessments at multiple points in the processing pipeline. This is particularly true when analyzing datasets with large numbers of subjects, coming from multiple investigators and/or institutions. These quality control procedures should monitor not only the quality of the original and processed data, but also the accuracy and consistency of acquisition parameters. Between-site differences in acquisition parameters can guide the choice of certain processing steps (e.g., resampling from oblique orientations, spatial smoothing). Various quality control metrics can determine what subjects to exclude from the group analyses, and can also guide additional processing steps that may be necessary. This paper describes a combination of qualitative and quantitative assessments to determine the quality of fMRI data. Processing is performed using the AFNI data analysis package. Qualitative assessments include visual inspection of the structural T1-weighted and fMRI echo-planar images, functional connectivity maps, functional connectivity strength, and temporal signal-to-noise maps concatenated from all subjects into a movie format. Quantitative metrics include the acquisition parameters, statistics about the level of subject motion, temporal signal-to-noise ratio, smoothness of the data, and the average functional connectivity strength. These measures are evaluated at different steps in the processing pipeline to catch gross abnormalities in the data, and to determine deviations in acquisition parameters, the alignment to template space, the level of head motion, and other sources of noise. We also evaluate the effect of different quantitative QC cutoffs, specifically the motion censoring threshold, and the impact of bandpass filtering. These qualitative and quantitative metrics can then provide information about what subjects to exclude and what subjects to examine more closely in the analysis of large datasets.

## Introduction

Functional MRI (fMRI) signal changes are relatively small and sensitive to various sources of noise, such a scanner artifacts, head motion, and other physiological fluctuations. Generating functional activation or connectivity maps from the acquired data therefore typically consists of a number of processing steps aimed at reducing this noise and aligning the brain images into a common space for group-level analyses. The programs used to perform this processing can vary between research groups, and each step often has multiple options that can be chosen by the researcher. An integral part of this processing pipeline is quality control (QC) to determine what processing steps or options are needed, to determine the source of any problems in the pipeline, to determine whether a subject should be excluded from group-level analyses, and ultimately to ensure the accuracy and validity of the final results.

Quality control should ideally be performed first in real-time, while the subject is being scanned and still in the MRI scanner. The advantage to this is that corrupted data can be immediately identified and then re-acquired or otherwise addressed. It is also critical to perform QC at multiple stages during the pre-processing. This QC can be both qualitative and quantitative. Qualitative measures, such as viewing the data at different stages during the processing, is extremely useful because of the myriad ways that the data can be corrupted or that the processing can go awry. A trained researcher can then determine what additional processing steps may be needed or what options or parameters should be adjusted. Quantitative measures of QC, such as the signal-to-noise ratio or the amount of head motion, are also important, particularly for large datasets where qualitative QC can be time consuming. These quantitative measures also allow for more reproducible analyses and inform the level of confidence in the final imaging results.

The most common problems affecting the quality of resting-state functional MRI data include imaging artifacts, subject head motion, and errors in aligning the data to a common template space. Imaging artifacts can include B0-field distortions or malfunctions in the RF coil leading to spikes or variations of signal intensity near malfunctioning coil elements. Head motion is common in fMRI and has long been recognized as a problem that needs to be minimized and reduced (Friston et al., 1996). Resting-state functional connectivity studies are particularly sensitive to the effects of motion since connectivity is measured by the temporal similarity of fMRI time series between two or more regions using some metric, such as the Pearson's correlation coefficient (Biswal et al., 1995). Two regions with correlated non-neuronal signal variations (noise) would result in an erroneously inflated functional connectivity, while two regions with uncorrelated noise would result in reduced connectivity. Even small amounts of motion can have significant impact on functional connectivity (Power et al., 2012; Satterthwaite et al., 2012; Van Dijk et al., 2012). Alignment of the functional data requires both the alignment of the T2^*^-weighted EPI to the T1-weighted structural image and the alignment of the T1-weighted structural to the template. The alignment between the EPI and T1 needs to take into account the differences in contrast between a T1-weighted and a T2^*^-weighted image. Alignment of the T1 to template space can involve non-linear transformations (e.g., image warping), and the accuracy of these depends of the quality of the removal of non-brain tissue (“brain extraction” or “skull-stripping”). Finally, problems can occur due to user error, such as prescribing an imaging volume that misses part of the brain or making an error in converting between file formats.

This paper provides several suggested QC procedures and measures for the analysis of resting-state functional MRI. This QC consists of both qualitative and quantitative measures, which are described in detail in the Methods section, and are applied to T1-weighted structural and resting-state functional MRI data provided by the OpenQC project. Finally, a determination is made whether to include or exclude each participant from further analyses, or when the inclusion or exclusion is borderline or depends on other factors.

## Methods

### MRI data

The MRI data consisted of T1-weighted structural MRI scans and T2^*^-weighted echo-planar imaging (EPI) resting-state functional MRI scans from 139 subjects drawn from 7 different sites, provided by the OpenQC project. These data were drawn from various publicly available MRI data repositories—ABIDE, ABIDE-II (Di Martino et al., 2014), Functional Connectome Project (Biswal et al., 2010), and OpenNeuro (Markiewicz et al., 2021). The EPI datasets all had a single echo time and did not have simultaneous multi-slice acquisitions. B0-field inhomogeneity measures (e.g., B0-field maps or EPIs with reversed phase encoding) were not provided.

### Processing pipeline

All data processing was performed using AFNI unless otherwise indicated (Cox, 1996). Processing scripts are available on GitHub: https://github.com/rbirn/OpenQC. The ICBM 152 non-linear atlas version 2009 was used as the template “MNI” brain (Fonov et al., 2011). The T1-weighted image volume was aligned to the MNI template by removing non-brain tissue signals and non-linearly warping the image to the template (using AFNI's @*SSwarper*). The T1-weighted image was segmented into gray matter, white matter, and CSF using FSL's *fast* (Zhang et al., 2001). The functional MRI echo-planar imaging (EPI) data were processed by first removing the first 4 volumes to assure that the magnetization is at steady-state. The data were then corrected for slice-timing differences (*3dTshift*), rotated and resampled to remove any oblique orientation (*3dWarp*), and registered to the first volume in each time series to reduce the effects of head motion (*3dvolreg*). B1-field inhomogeneities (bias field) were estimated using *N4BiasFieldCorrection* from ANTs (Tustison et al., 2010). The data were then divided by this bias field to correct for B1-field inhomogeneity. The echo-planar image was aligned to the T1-weighted structural scan using a 12-parameter affine transformation (*align_epi_anat.py*). The EPI-to-T1 and T1-to-template transformations were then concatenated and used to non-linearly warp the fMRI data to the MNI template. In order to further reduce the effects of physiological noise and head motion, several nuisance regressors were included in a general linear model and projected out of the data (*3dTproject*). These included the average signal over the whole brain, the average signal over eroded white matter, average signal over CSF, the 6 realignment parameters, and the temporal derivatives of each of these regressors. This general linear model also included 2 polynomials (to account for slow drifts) and a set of sines and cosines to band-pass filter the data from 0.01 to 0.1 Hz. Time points where the volume-to-volume motion exceeded a predefined motion censoring threshold, as well as the preceding time points, were excluded (censored) from the nuisance regression. Three different motion censoring thresholds were evaluated: 0.2, 0.4, 1.0 mm. Prior studies have shown that one source of variability in multi-site studies are differences in the spatial smoothness of the data (Friedman et al., 2008). Since the data in this study were acquired at different sites and different spatial resolutions, rather than applying a fixed amount of spatial smoothing, the data were then iteratively smoothed to a achieve a final full-width at half maximum (FWHM) of 8 mm (using *3dBlurToFWHM*). For comparison, the data processing was repeated without regressing out the average whole-brain signal (global signal regression, GSR).

Functional connectivity maps were generated for 4 seed regions of interest—4 mm radius spheres located in the posterior cingulate (MNI coordinate: 0, 50, 31), the left primary motor cortex (MNI coordinate: 36, 20, 60), left auditory cortex (MNI coordinate: 43, 25, 14), and the left primary visual cortex (MNI coordinate: 30, 87, 9). These seed regions identify the default mode network, motor network, auditory network, and visual network, respectively. The preprocessed signal was averaged over each seed region of interest, and the Pearson's correlation coefficient between this seed time course and all other voxel time courses was computed. In addition to these voxel-wise functional connectivity maps, connections between multiple regions across the whole brain was investigated by computing a functional connectivity matrix. The brain was divided into 333 regions of interest according to a parcellation by Gordon et al. (2016). The preprocessed signal was averaged over each region of interest, and all pairwise correlations were computed.

For comparison of QC metrics, data were also processed using the more automated pipeline provided with AFNI, *afni_proc.py*. This pipeline used as input the original resting-state EPI and the T1 processed (brain extracted and aligned to template space) by @SSwarper, and included the following processing steps: removal of first 4 time points; alignment of EPI to T1; volume registration (motion correction); non-linear warping to template space; nuisance regression using average signal over eroded white matter and CSF, motion, and their derivatives; band-pass filtering (0.01–0.1 Hz); and blurring to a FWHM of 8 mm. This pipeline by default derives a set of quality control metrics from each subject and assembles them into an html-formatted document that can be viewed in a web browser.

### Quality control procedures

First, several imaging parameters were extracted from the data and compared. This included the spatial resolution (voxel size), matrix size, repetition time (TR), obliquity, and number of time points (image volumes) acquired. These values informed some of the processing choices and QC criteria. Specifically, the fact that data were acquired at different spatial resolutions motivated iterative blurring of the data to a final resolution rather than applying a fixed spatial blur across subjects. The observation that some data were acquired with oblique orientations necessitated that this be accounted for when registering the EPI to the T1-weighted structural scan and the T1-weighted structural to the template. The total number of time points acquired needs to be considered when applying certain QC criteria (e.g., the total number of “good” time points). The imaging parameters were also examined for any deviations from other scans acquired at that site. The processing pipeline described above was then run on each dataset. Log files were generated that contained any status or error messages (typically output to the screen). These log files were examined when the processing pipeline failed to produce the final preprocessed data output.

The image quality and alignment of each subject's T1-weighted structural scan to template space was examined by concatenating the T1-weighted images across subjects. Similarly, a single echo-planar image volume, after warping to template space but before nuisance regression or spatial smoothing, was extracted from each subject and concatenated across subjects. These series of image volumes were then be played as a movie within the AFNI GUI to identify any misalignments or other imaging artifacts. Functional connectivity maps for each of the seed regions were similarly concatenated and played as a movie, with the subject's T1-weighted image as the underlay and the functional connectivity as an overlay.

### QC metrics

A number of quantitative metrics were computed, using the first (non afni_proc.py) pipeline described above, to assess data quality. These are briefly described below.

#### Left-right flip

Potential errors in the left-right orientation (i.e., accidental flips of the data in the L-R direction) were investigated by flipping the structural T1 dataset in the left-right direction and repeating the alignment between the EPI and T1. This is performed using the -check_flip option in AFNI's *align_epi_anat.py*. If the cost function for the alignment is lower for the flipped dataset, either the EPI or T1 is likely flipped in the L-R direction.

#### FWHM

The smoothness of the acquired EPI data were determined by computing the full-width at half-maximum (FWHM) in each of the 3 cardinal directions (using 3dFWHMx). This measure can be used to determine whether variations in the image matrix are due to differences in the acquisition (e.g., acquiring data at a higher resolution) or to differences in the processing (e.g., resampling the data to a higher resolution). This information can then guide other processing choices, such as the amount of smoothing to apply, or whether to smooth to a predetermined amount of smoothness.

#### Temporal signal-to-noise ratio (TSNR)

The temporal signal to noise ratio was computed by dividing the mean signal over time in each voxel of the original acquired image by its standard deviation over time. This measure can be good at identifying data severely corrupted by head motion, RF coil problems (e.g., spiking), or other imaging artifacts. This measure does vary with the imaging parameters (resolution, number of averages, parallel imaging acceleration, field strength, echo time, etc.), so it is difficult to set a strict cutoff. However, the average TSNR over the whole brain can be compared to other subjects within the group acquired with similar imaging parameters at that site.

#### Mean Enorm

Volume-to-volume head motion was assessed by first computing the temporal difference of each image realignment parameter (3 translations, 3 rotations), and then computing the Euclidean norm (square-root of the sum of squares, Enorm) of these temporal differences at each time point, with shifts in millimeters and rotations in degrees. Note that a 1 degree rotations corresponds to a displacement of 1 mm at a radius of 57 mm, roughly the distance from the center of mass to the edge of the brain. The mean value of the Enorm across time provides a measure of the mean (average) volume-to-volume motion for that imaging run.

#### Max Enorm

The maximum of the Enorm time course (computed as described above) across time provides a measure of the maximum motion from one time point to the next. The rationale for excluding subjects based on the maximum motion is that large motion is more likely to be associated with changes in B0-field distortions, moving into different parts of the RF coil sensitivity, and spin-history effects. However, if large motion is infrequent, there are approaches to reduce the resultant signal changes (Birn et al., 2022).

#### Number of “good” time points

The number of time points remaining after censoring time points exceeding a certain motion (Enorm) threshold. A related, and from a quality control viewpoint equivalent, metric is the degrees-of-freedom remaining after censoring, band-pass filtering, and nuisance regression. Enough degrees-of-freedom should remain to accurately estimate the functional connectivity. A degree-of-freedom cutoff of 15 was used for this study. Studies have also shown that the specificity (Van Dijk et al., 2010), test-retest reliability (Birn et al., 2013) and the identification accuracy (Finn et al., 2015) of functional connectivity increases with both greater number of time points and duration of acquisition. A QC cutoff of at least 5 min of good data has been used by prior studies (Van Dijk et al., 2010; Power et al., 2014, 2015). However, 3 of the sites in this study acquired only 5 min of data or less. Therefore, a QC cutoff of 4 min was used for this study.

#### Dice_e2a

The Sorensen-Dice coefficient between the echo-planar fMRI brain image and T1-weighted structural is computed as two times the intersection between whole-brain masks of the echo-planar image and T1-weighted image (after alignment, in template space) divided by the sum of the areas of each of these masks. The goal of this metric is to measure the accuracy of the EPI-to-T1 alignment. This measure can be computed using the AFNI program *3ddot*.

#### Dice_a2t

The Sorensen-Dice coefficient between the T1-weighted structural and MNI template is computed similar as above, but with whole-brain masks of the T1-weighted and MNI template images. The goal of this metric is to measure the accuracy of the T1-to-template alignment.

#### FCS

The functional connectivity strength (FCS) is the average functional connectivity from each voxel to all other voxels in the brain. Mathematically this is identical to computing the correlation between each voxel time series and a scaled version of the global signal. This scaled version of the global signal is computed by dividing each voxel's signal intensity time course by its standard deviation over time, and then computing the average of these scaled signals over the entire brain. This metric can be used to identify abnormally high correlations that may result from some RF coil problems, for example a loose connection in one of the coil elements causing spikes in the signal. These signal spikes occur at the same time across large portions of the image thus causing the time courses to be highly correlated. The rationale for using this measure in addition to TSNR is that a single spike may not affect the TSNR very much, but can affect the correlation of that voxel time course with all other voxel in that slice.

#### Similarity to mean FC

The similarity of the mean functional connectivity is determined by computing the correlation between each subject's functional connectivity matrix and the group average functional connectivity matrix (using AFNI's *3ddot*). This metric can identify potential outliers in functional connectivity. For comparison, the similarity was also using the Euclidean distance between each subject's functional connectivity matrix and the group mean functional connectivity matrix. To distinguish this metric from the similarity using Pearson's correlation, we call this the “Dissimilarity” since a greater Euclidean distance is associated with a reduced similarity and thus greater dissimilarity. This was computed using AFNI's *3dcalc* and *3dROIstats*.

### Determination of QC criteria

A common QC criterion is to exclude time points whose framewise displacement (volume-to-volume motion) exceeds 0.2 mm (Power et al., 2014, 2015). We wanted to examine whether this censoring threshold was appropriate for the current study. Therefore, the processing pipeline was run for 3 different motion censoring thresholds: 0.2, 0.4, and 1.0 mm. In addition, we compared the functional connectivity both with and without bandpass filtering.

One measure that has been used to assess the effectiveness of different processing choices is the correlation between the functional connectivity and a quality control metric, such as the mean Enorm—a measure referred to as QC-FC (Ciric et al., 2018). This is essentially testing whether there is a difference in functional connectivity as a function of head motion, i.e., between high-motion and low-motion subjects. We therefore computed the correlation between the functional connectivity and the mean Enorm for each connection in the connectivity matrix. We then computed a histogram of these correlation values. An additional metric that has been used to evaluate the effectiveness of different processing choices is the distance dependence of motion artifacts (Power et al., 2012, 2014, 2015; Ciric et al., 2018). This is computed as the correlation between the QC-FC metric described above and the distance between each of the nodes in the connectivity matrix.

We also looked at the similarity of each subject's functional connectivity matrix to the group average functional connectivity matrix, as described above. We then examined the correlation of this similarity with motion, specifically the mean Enorm. The rationale for the motion censoring threshold that we chose is provided in the results section (below).

### Resources

The following software packages and versions were used in the analysis:

AFNI Version AFNI_21.2.07 (precompiled binary linux_openmp_64, Sep. 20, 2021).
FSL Version 6.0.4.
ANTs Version 0.0.0 (compiled May 26, 2020).

## Results

The set of quality control (QC) summary criteria used for excluding or identifying problematic subjects in this study are shown in Table 1. The quality control procedures identified a number of problems with the data, leading to the exclusion of some of the subjects and modified processing for others. Very similar results were obtained from the *afni_proc.py* and our custom AFNI pipeline.

**Table 1:** QC criteria summary table.

**Resting state fMRI QC criteria: Exclude (or re-examine) a subject if:**
(A) Fewer than 15 degrees-of-freedom are left after motion censoring, nuisance regression, and band-pass filtering
(B) Fewer than 4 min (240 s) of data remain after motion censoring
(C) Maximum Enorm (volume-to-volume motion) > 3 mm
(D) The data are left-right flipped and the correct orientation cannot be determined
(E) Temporal signal-to-noise and/or FCS indicate the presence of an RF coil artifact (e.g., spiking)
(F) Part of the cortex is out of the field of view (qualitative)
(G) There are large abnormalities in the anatomy (qualitative)
(H) There are significant mis-alignments in the data to template space that cannot be fixed with different processing choices (qualitative)

Examination of the imaging parameters showed that some of the datasets were acquired (or reconstructed) at a different matrix size compared to others from the same site. For example, sub-118 had a matrix size of 112 voxels while all other scans from that site had a matrix size of 96 voxels. The json files associated with the data all indicate that the data from this site was acquired with a matrix size of 84 × 81. For site 5, 15 subjects had a matrix size of 80 voxels while 5 subjects had a matrix size of 128 voxels. The datasets from this site with 128 voxels had significantly greater smoothness (FWHM in the x- and y-directions) compared to the datasets with 80 voxels (*p* < 0.004), suggesting that the data was re-interpolated after acquisition, resulting in increased blurring.

**Figure 1:**
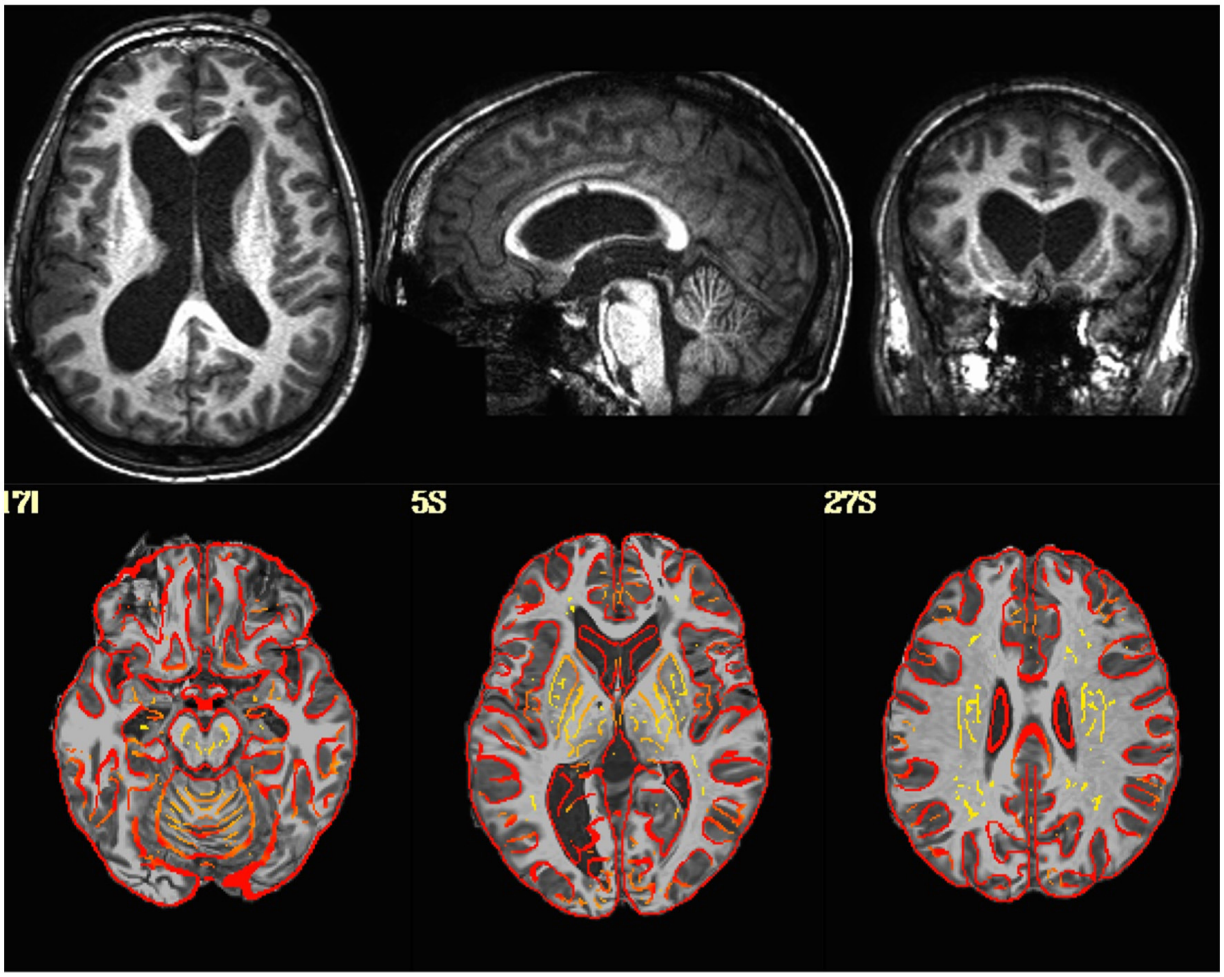
**(Top Row)** T1-weighted image in native space for a subject with enlarged ventricles. **(Bottom Row)** T1-weighted image non-linearly aligned to template space as underlay, with the gray/white matter boundaries from the template brain overlayed in red. Dice coefficient between the subject's T1 and the template = 0.96.

Visualization of the original EPI datasets indicated that two datasets (sub-518, sub-519) were upside down, with the I-S axis inverted. Alignment between the EPI and T1 indicated that two subjects (sub-101, sub-115) had either the EPI or T1 flipped in the L-R direction. Visualization of the T1-weighted structural images indicated that one subject (sub-509) had much larger ventricles than the rest of the sample (Figure 1).

**Figure 2:**
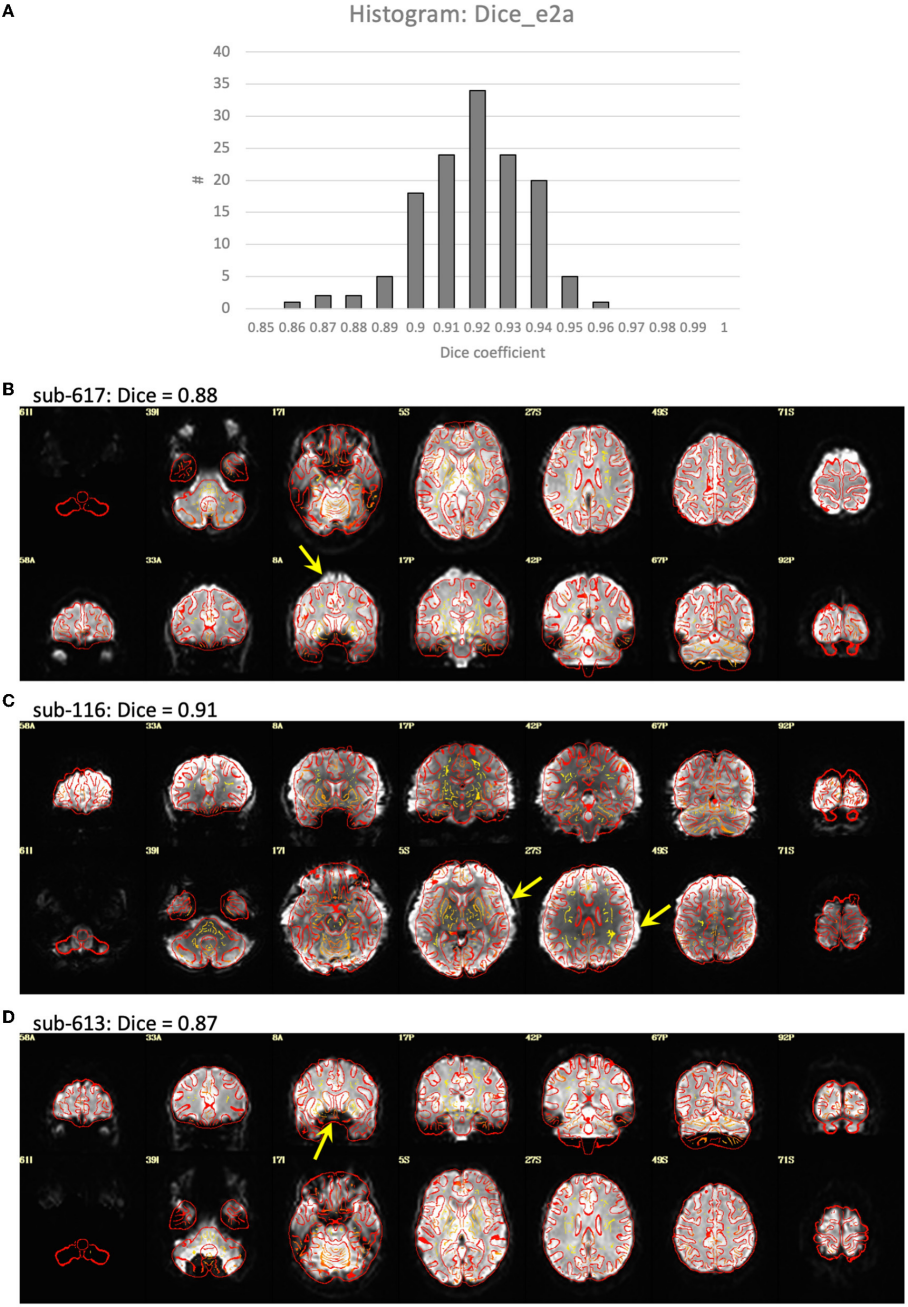
Alignment between the EPI and T1-weighted structural image. **(A)** Histogram of the Dice coefficients of the EPI and anatomical T1-weighted brain masks (Dice_e2a). **(B–D)** Case examples of the alignment between the EPI (in grayscale) and T1 (in red outline). **(B)** Subject 617 shows a slight misalignment between the EPI and T1 in the superior region of the brain (yellow arrow), and has a relatively low Dice coefficient = 0.88 compared to the rest of the group. **(C)** Subject 116 shows a slight misalignment, a stretching of the EPI in the left-right direction (yellow arrows), but has a Dice coefficient close to the mean of the group, Dice = 0.91. **(D)** Subject 613 shows a good alignment between the EPI and T1 in the cortex, but has a signal dropout in the frontal lobe resulting in a relatively low Dice coefficient = 0.87.

Visualization of the T1-weighted images concatenated across subjects and played as a movie indicated good alignment of each T1 to the template. Alignment of the EPI to template space was generally quite good, but had a greater variability across subjects with some brain areas showing a slight misalignment to the template brain in some subjects (Figure 2). Closer examination of the processing in these subjects indicated that this misalignment to template space was due to a poor alignment between the EPI and T1-weighted image, even after automatic alignment. The Dice coefficient between the EPI and T1 (Dice_e2a) was lower for some of the misaligned participants compared to the rest of the group. However, some participants had lower Dice coefficients due to B0-field inhomogeneity induced signal dropout, and other subjects had Dice coefficients close to the group mean despite showing substantial misalignments (Figure 2).

**Figure 3:**
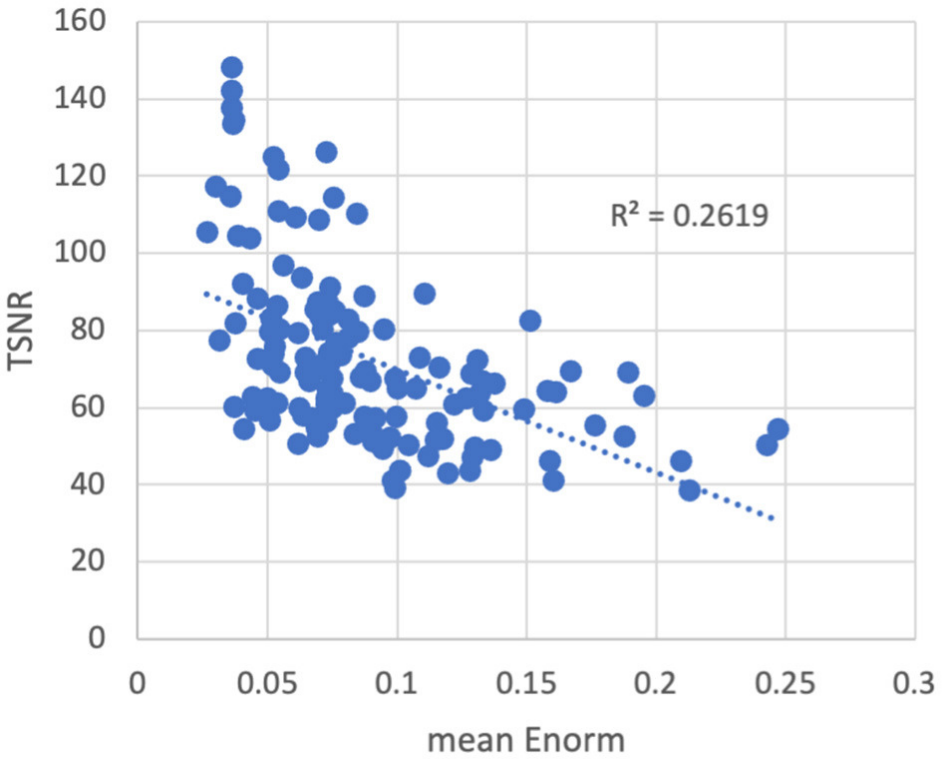
Temporal signal-to-noise ratio (TSNR) vs. the mean volume-to-volume motion as measured by the Euclidean norm (Enorm) of the temporal difference of the 6 realignment parameters. As motion increases, the TSNR decreases. Note that subjects with higher motion have lower TSNR, but the converse is not necessarily true—subjects with low motion can also have low TSNR, possibly due to other non-motion sources of noise.

As expected, temporal signal-to-noise ratio (TSNR) was reduced in subjects with higher amounts of motion (Figure 3). The converse was not necessarily true—some subjects with low motion also had low TSNR, possibly due to other non-motion sources of noise. No outliers or abnormalities were found in the temporal SNR or functional connectivity strength to indicate any coil artifacts. Similarly, the entire cortex was scanned in all subjects.

**Figure 4:**
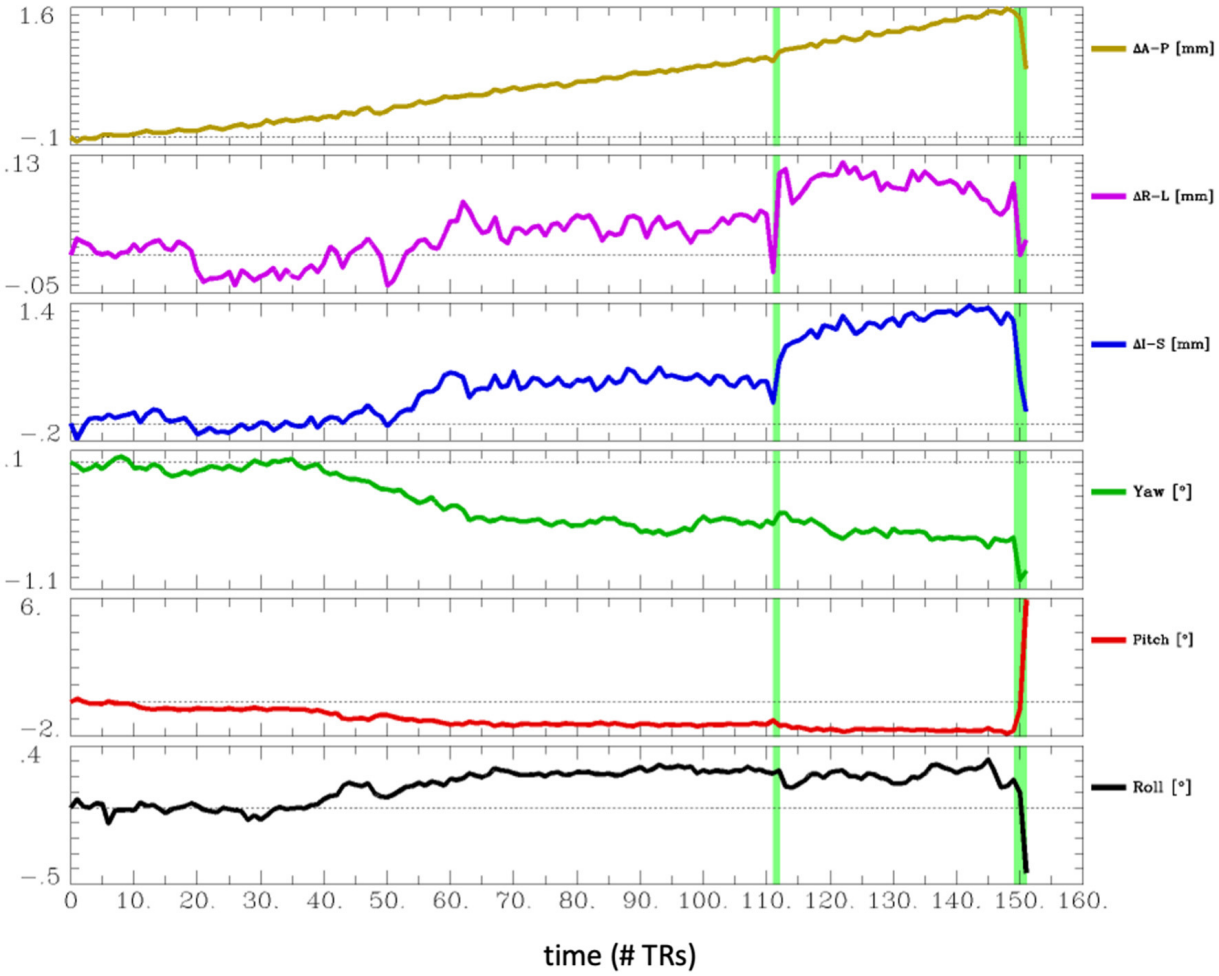
Estimated head motion realignment parameters for subject sub-102, which had the largest maximum volume-to-volume motion of 6.5 mm. However, this motion occurred at the end of the run, so the effects of this motion can be eliminated by censoring the last few time points.

The most common problem across datasets was excessive head motion. At an Enorm censoring threshold of 0.2 mm, 15 subjects did not have enough degrees of freedom left for the nuisance regression and bandpass filtering. A total of 26 subjects had very low degrees of freedom (<15), and 16 subjects had <4 min of data left after censoring. At a censoring threshold of 0.4 mm, 2 subjects did not have enough degrees of freedom after censoring, 4 subjects had very low degrees of freedom, and 2 subjects had <4 min of data left after censoring. Two subjects had one or more movements >3 mm. A closer examination of the subject with the largest motion of 6.5 mm (sub-102) revealed that the motion occurred right at the end of the imaging run (Figure 4). The effect of this motion can therefore be eliminated by censoring the time points at the end of the imaging run.

### Rationale for QC criteria: Motion censoring threshold

**Figure 5:**
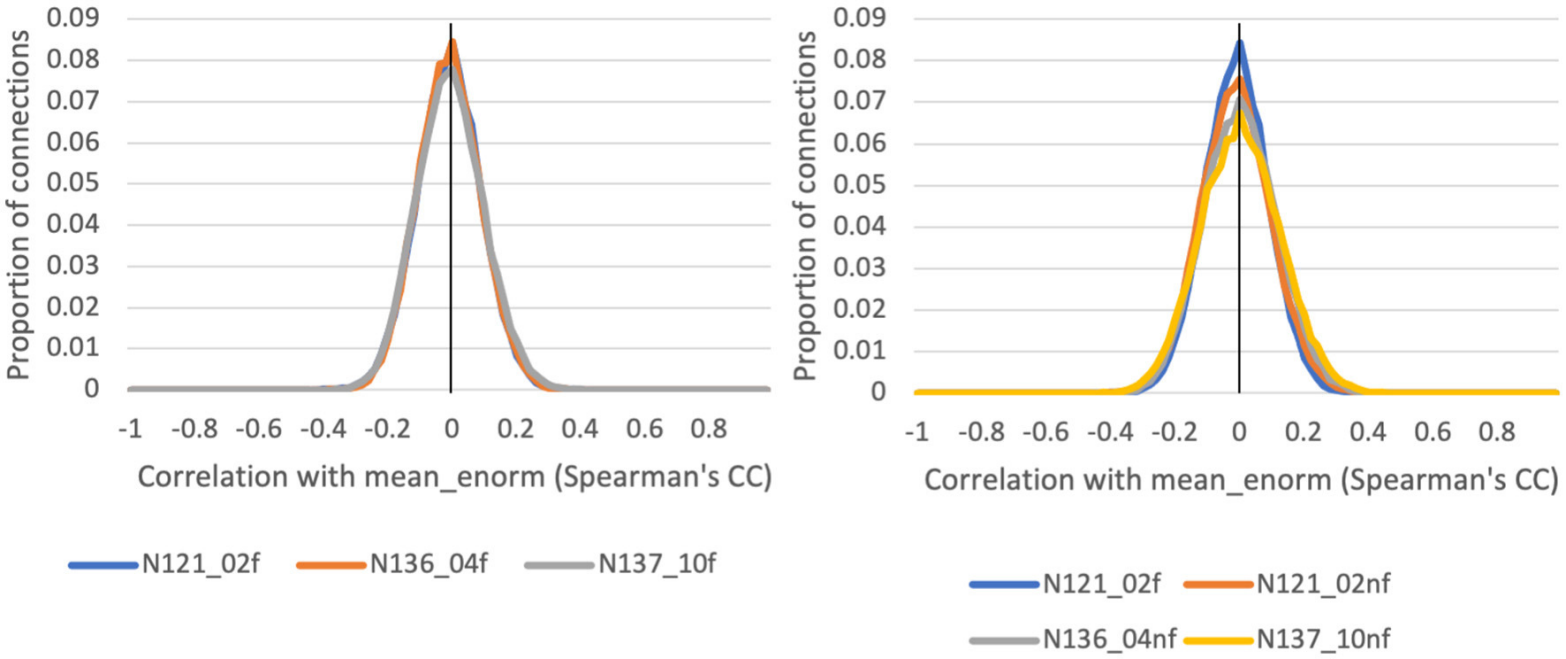
Histograms of the correlation between a quality control (QC) criterion—the mean Enorm—and the functional connectivity (FC): QC-FC, for 3 different motion censoring thresholds (02 = 0.2 mm, 04 = 0.4 mm, 10 = 1.0 mm) with (f) and without (nf) temporal bandpass filtering (0.01–0.1 Hz).

The correlation between functional connectivity and mean Enorm (QC-FC) was highly similar for censoring thresholds of 0.2, 0.4, and 1.0 mm (Figure 5). The mean correlation of FC with motion was close to zero (0.00001 for a motion censoring threshold of 0.2 mm, 0.001 for censoring threshold 0.4 mm, and 0.004 for a censoring threshold of 1.0 mm). The histogram showed slightly wider tails, indicating some connections with greater correlation with motion, at a censoring threshold of 1.0 mm compared to 0.4 or 0.2 mm. The QC-FC was slightly increased when no bandpass filtering was performed. There was very little distance dependence of the QC-FC. At a motion censoring threshold of 0.2 mm, the correlation between QC-FC and distance was −0.004 (95% confidence interval: −0.012 to 0.004). At a motion censoring threshold of 0.4 mm the distance dependence correlation was −0.0009 (−0.009, 0.007), and at a motion censoring threshold of 1.0 mm the correlation was 0.005 (−0.003, 0.013).

**Figure 6:**
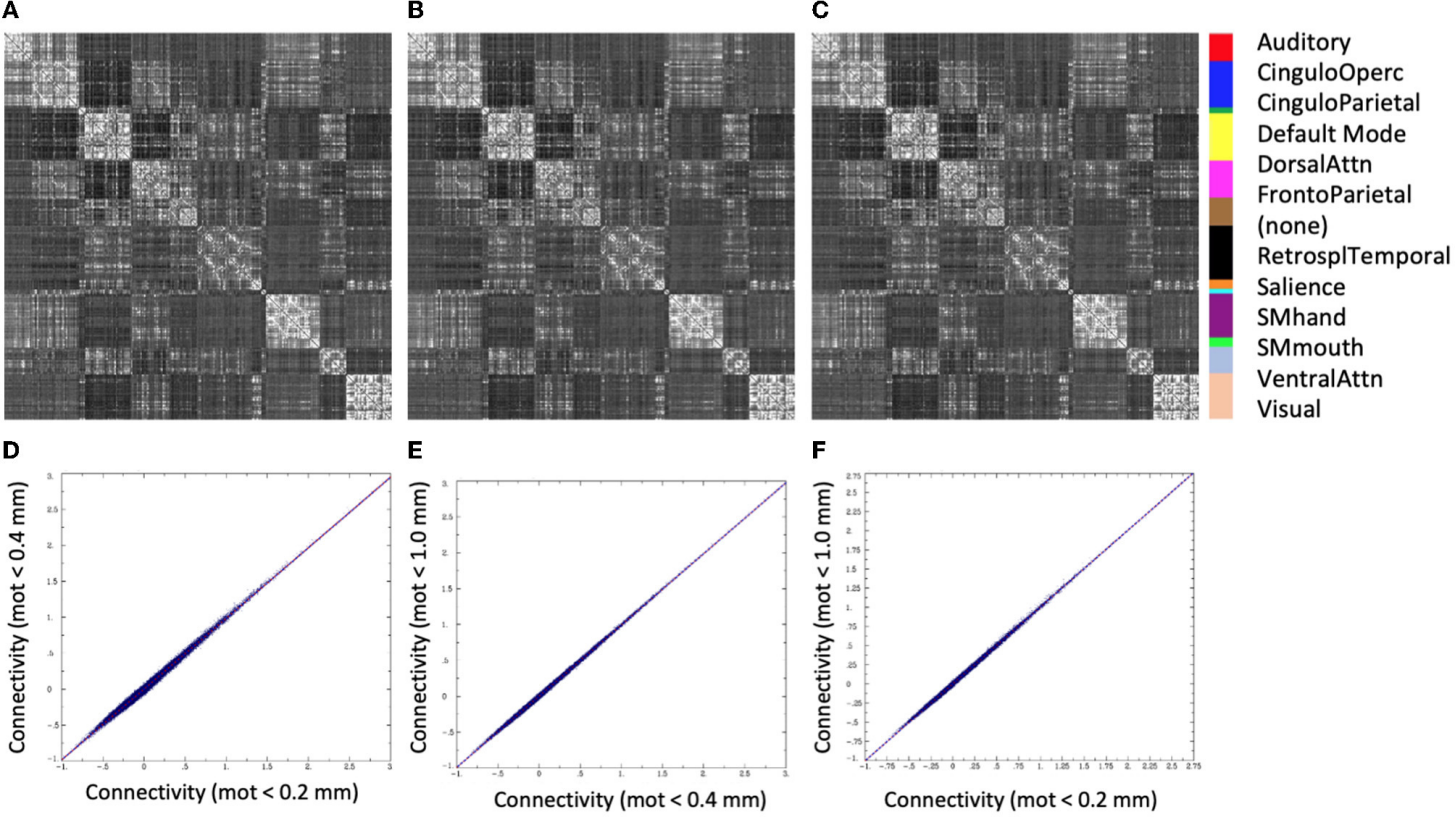
Group average functional connectivity matrices for data with different motion-censoring thresholds: **(A)** volume-to-volume motion (Euclidean norm, Enorm) < 0.2 mm, **(B)** Enorm < 0.4 mm, **(C)** Enorm < 1.0 mm. **(D–F)** Connectivity values (Fisher-Z transformed correlation coefficients) for **(D)** Enorm < 0.2 mm vs. Enorm < 0.4 mm, **(E)** Enorm < 0.4 mm vs. Enorm < 1.0 mm, **(F)** Enorm < 0.2 mm vs. Enorm < 1.0 mm. Group average matrices are highly similar for these 3 different levels of motion censoring.

**Figure 7:**
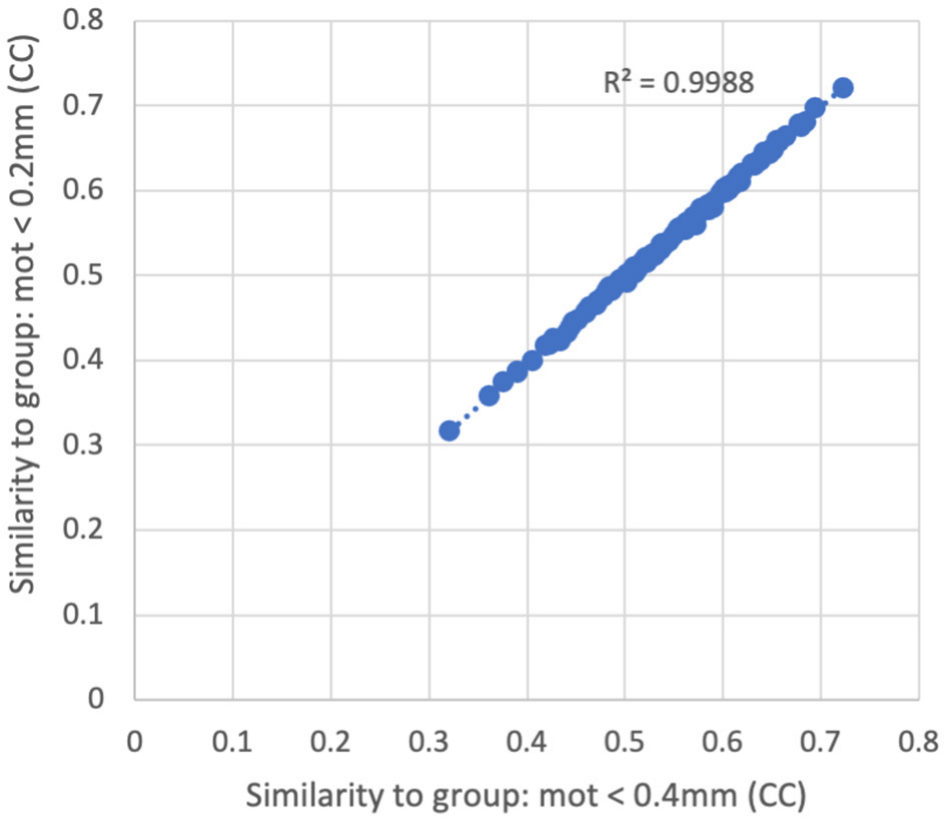
Similarity of each subject's functional connectivity matrix (using a censoring threshold of 0.4 mm) to the group average functional connectivity matrix that used either 0.2 mm (y-axis) or 0.4 mm (x-axis) censoring threshold. The similarity is nearly identical (*R*^2^ = 0.9988) regardless of which threshold was used in the formation of the group maps.

There was very little difference in the group functional connectivity matrices using censoring thresholds of 0.2, 0.4, or 1.0 mm (Figure 6). The similarity of each subject's functional connectivity to group mean functional connectivity was nearly the same whether the group functional connectivity matrix was formed using 0.2 vs. 0.4 mm censoring thresholds (*R*^2^ = 0.999) (Figure 7). Therefore, it does not matter which motion threshold was used as the group functional connectivity for comparison in computing the similarity.

**Figure 8:**
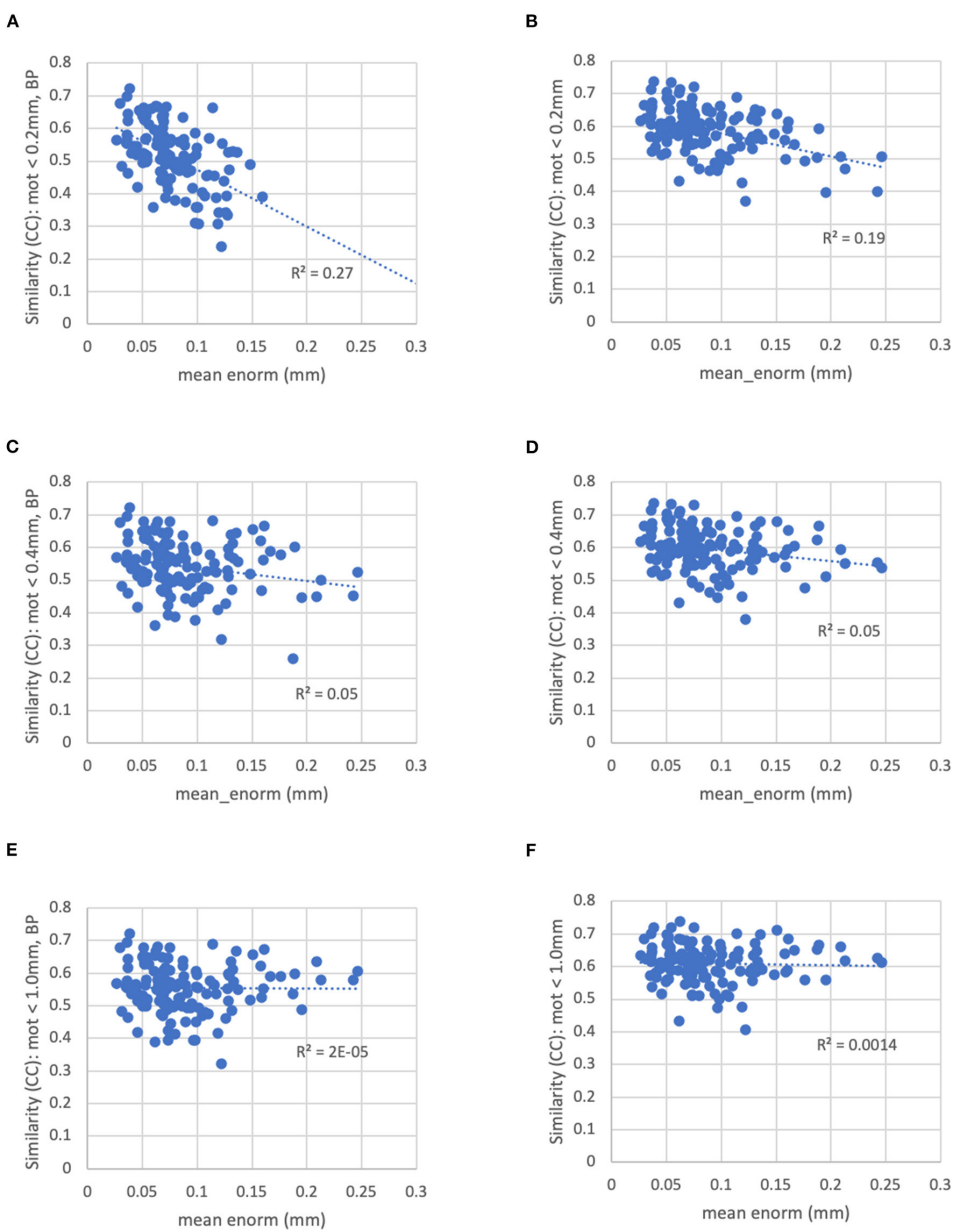
The similarity between each subject's functional connectivity matrix and the group-average functional connectivity matrix for different motion censoring thresholds (0.2, 0.4, 1.0 mm) with and without bandpass filtering (BP). BP, bandpass filtering (0.01–0.1 Hz), no BP, no bandpass filtering. **(A)** At a motion censoring threshold of 0.2 mm with bandpass filtering, subjects with higher motion (mean Enorm) show reduced similarity (*R*^2^ = 0.27). **(B)** Without bandpass filtering, similarity is increased, but subjects with higher motion still show lower similarity (*R*^2^ = 0.19). **(C)** At a motion censoring threshold of 0.4 mm with bandpass filtering, similarity to the group mean connectivity is only weakly correlated with motion (*R*^2^ = 0.05). **(D)** Without bandpass filtering, there is again only a weak correlation with motion. **(E)** At a motion censoring threshold of 1.0 mm and bandpass filtering, there is very little correlation between the similarity and motion (*R*^2^ = 0.00002). **(F)** Without bandpass filtering at a motion threshold of 1.0 mm, there is very little correlation with motion across subjects (*R*^2^ = 0.0014).

**Figure 9:**
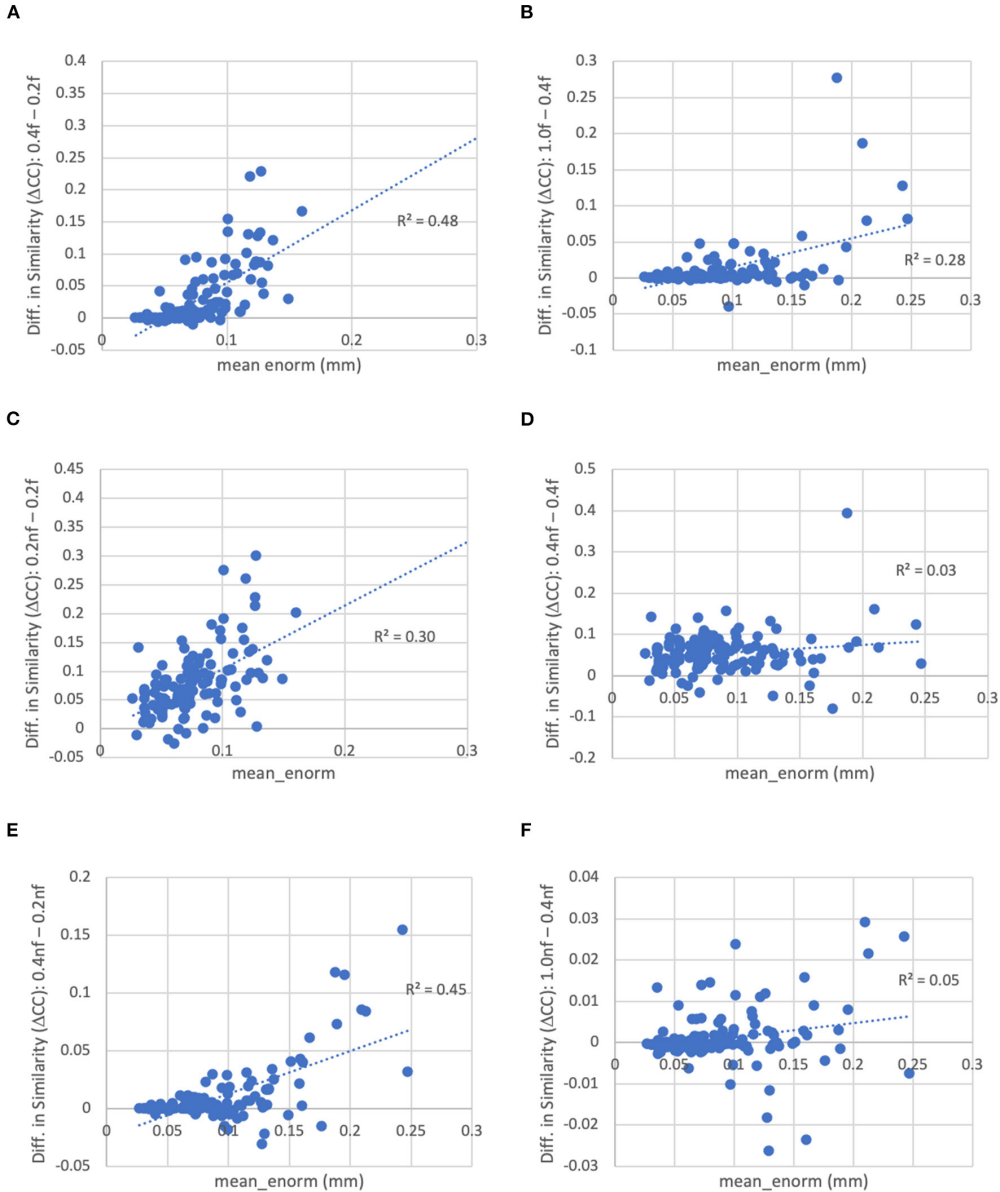
Difference in the similarity of each subject's functional connectivity matrix to the group mean for different levels of motion censoring, with (f) and without (nf) bandpass filtering. **(A)** With bandpass filtering, similarity is increased for connectivity matrices computed at a motion threshold of 0.4 vs. 0.2 mm, particularly in subjects with high motion. **(B)** Similarly with bandpass filtering, similarity is increased for a motion censoring threshold of 1.0 mm compared to 0.4 mm, particularly for high-motion subjects. **(C)** At a motion-censoring threshold of 0.2 mm, not performing bandpass filtering increases the similarity compared to performing bandpass filtering, particularly in high-motion subjects. **(D)** At a motion-censoring threshold of 0.4 mm, similarity to the group-mean is increased for most subjects without vs. with bandpass filtering, but less dependent on the mean level of motion. **(E)** Without bandpass filtering, a motion censoring threshold of 0.4 mm has greater similarity than a threshold of 0.2 mm, particularly for high-motion subjects. **(F)** Without bandpass filtering, using a motion censoring threshold of 1.0 mm compared to 0.4 mm can result in either increases or decreases in similarity to the group mean, with little correlation to mean motion.

With a censoring threshold of 0.2 mm, the similarity was strongly dependent on the mean motion with lower similarity for subjects with higher motion (*R*^2^ = 0.27) (Figure 8A). However, at a motion censoring threshold of 0.4 mm, the similarity was only weakly related to subject motion (*R*^2^ = 0.04) (Figure 8C). The similarity of functional connectivity to the group mean was greater for a censoring threshold of 0.4 mm compared to 0.2 mm and this difference was greater in high-motion subjects (Figure 9A). That is, high-motion subjects had a higher similarity of their functional connectivity matrices to the group average using a 0.4 mm threshold compared to a more stringent 0.2 mm. This suggests that the decreased similarity in high-motion subjects at a 0.2 mm censoring threshold is due to the reduced degrees of freedom from aggressive time point censoring rather than corruption of the functional connectivity due to motion. Similarity was further increased, particularly in high-motion subjects, by using a motion-censoring threshold of 1.0 mm (Figure 9B). However, this threshold is much higher than is currently used in the field, and combined with the slightly higher correlation with motion (QC-FC) at a 1.0 mm censoring threshold, we decided to use a 0.4 mm censoring threshold as the cutoff.

**Figure 10:**
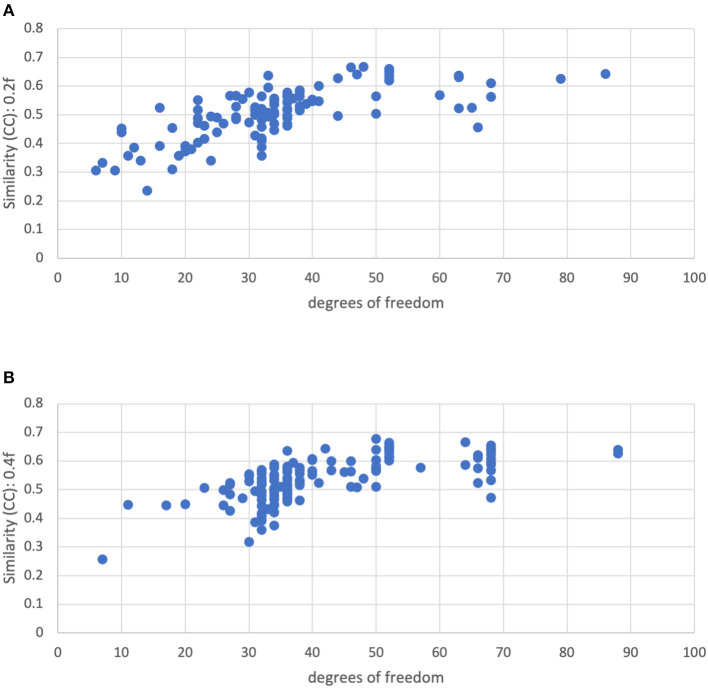
Similarity of each subject's functional connectivity matrix to the group mean for a motion censoring threshold of **(Top)** 0.2 mm and **(Bottom)** 0.4 mm, vs. the degrees of freedom left after motion censoring, bandpass filtering, and nuisance regression. The similarity appears to be roughly linearly related to the degrees of freedom for low degrees of freedom (<50), plateauing at higher degrees of freedom.

Figure 10 shows the similarity vs. degrees of freedom for a censoring threshold of 0.2 and 0.4 mm. Similarity is reduced for lower degrees of freedom. Moreover, there is no clear cutoff for the similarity at low degrees of freedom. The similarity appears to be roughly linearly related to the degrees of freedom for low degrees of freedom (<50), plateauing at higher degrees of freedom. We decided to use a cutoff of 15 degrees of freedom to reduce the influence of severe motion while still retaining enough subjects in the group analysis.

**Figure 11:**
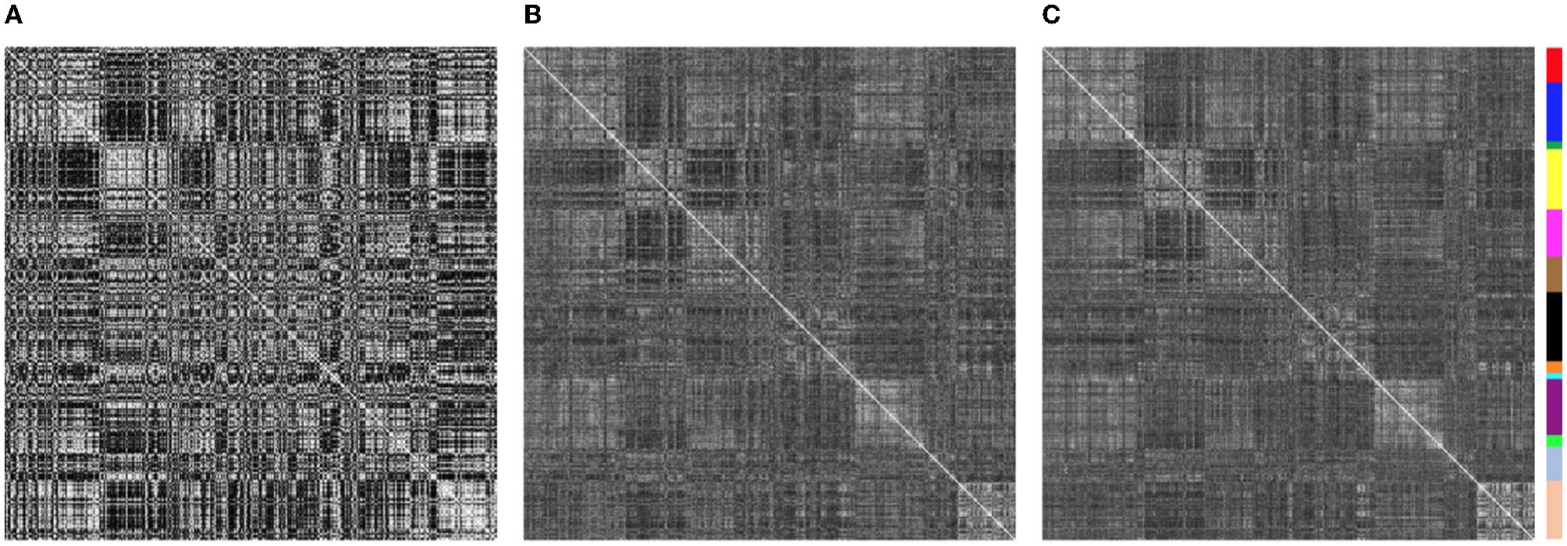
Functional connectivity matrices from sub-507. **(A)** With a motion censoring threshold of 0.4 mm, only 7 degrees of freedom are left, and matrix is quite noisy, quite different than the group mean functional connectivity matrix. **(B)** At a motion censoring threshold of 0.2 mm and no bandpass filtering, the functional connectivity matrix is more similar to the group average functional connectivity. **(C)** Connectivity matrix at a motion censoring threshold of 0.4 mm is very similar to a threshold of 0.2 mm when no bandpass filtering is applied.

**Figure 12:**
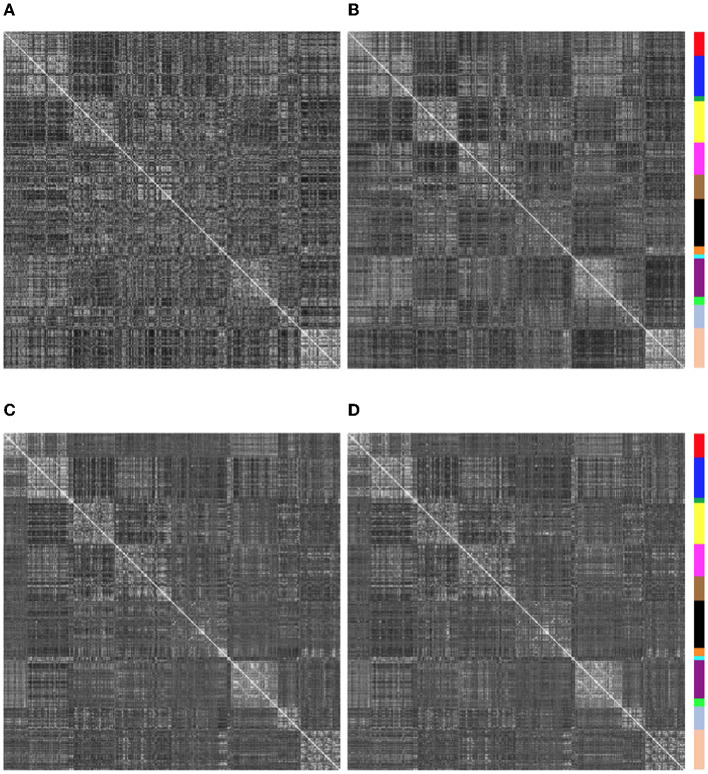
Functional connectivity matrices for 2 low-motion subjects with and without bandpass filtering (0.01–0.1 Hz). Degrees of freedom (dof) after motion censoring, nuisance regression, and with/without bandpass filtering are shown in the title. **(A, B)** Sub-501, mean Enorm = 0.03, no time points censored and 140 time points left at a censoring threshold of 0.4 mm. **(A)** With bandpass filtering, 32 degrees of freedom are left. **(B)** Without bandpass filtering, 119 degrees of freedom are left. Note that the pattern of within-network and between-network connectivity is noisier and less like the group average maps when bandpass filtering is applied. **(C, D)** Sub-606, mean Enorm = 0.03 mm, no time points censored and 720 time points left at a censoring threshold of 0.4 mm. **(C)** With bandpass filtering, 306 degrees of freedom are left. **(D)** Without bandpass filtering, 699 degrees of freedom are left. Functional connectivity matrices are highly similar with and without bandpass filtering since both have high degrees of freedom.

Similarity of functional connectivity to the group mean was also increased by eliminating the band-pass filtering step (see Figures 8, 9). One example of this is shown in Figure 11 for a subject (sub-507) that had only 7 degrees of freedom left after bandpass filtering and motion censoring with a threshold of 0.4 mm. This connectivity matrix appears quite noisy (Figure 11A) At a motion censoring threshold of 0.2 mm and no bandpass filtering, the functional connectivity matrix is more similar to the group average functional connectivity (Figure 11B). The connectivity matrix for this subject at a motion censoring threshold of 0.4 mm is very similar to a threshold of 0.2 mm when no bandpass filtering is applied (Figure 11C). Increase in similarity when eliminating the bandpass filtering step was observed even in low-motion subjects (Figure 12). Subject sub-501 had a mean Enorm of 0.03 mm with no time points censored at a threshold of 0.4 mm. Thirty-two degrees of freedom were left with bandpass filtering, and 119 degrees of freedom were left without bandpass filtering. The pattern of within-network and between-network connectivity was noisier and less like the group average maps when bandpass filtering was applied. Figures 12C, D shows the connectivity matrix from a low-motion subject (sub-606) that had longer time series (720 time points), with no time points censored at a threshold of 0.4 mm, 306 degrees of freedom left after bandpass filtering and 699 degrees of freedom without bandpass filtering. Functional connectivity matrices are highly similar with and without bandpass filtering since both have high degrees of freedom.

The similarity was further improved by relaxing the motion censoring from 0.2 to 0.4 mm (Figure 9E). That is, the increase in similarity for a motion censoring threshold of 0.4 vs. 0.2 mm, both without bandpass filtering, was greater in subjects with higher mean_enorm, again likely due to the greater degrees of freedom with a more relaxed censoring threshold. Without bandpass filtering, the similarity was slightly correlated with mean_enorm at a censoring threshold of 0.2 mm (*R*^2^ = 0.19, Figure 8B), but only weakly correlated with subject motion at a censoring threshold of 0.4 mm (*R*^2^ = 0.05, Figure 8D). The improvements in similarity with vs. without bandpass filtering was correlated with the mean_enorm at a censoring threshold of 0.2 mm (*R*^2^ = 0.30, Figure 9C) but not 0.4 mm (*R*^2^ = 0.03, Figure 9D). These results all suggest that the similarity is improved by not applying bandpass filtering and by using a less stringent censoring threshold (e.g., 0.4 mm) due to the increased degrees of freedom. Without bandpass filtering, using a motion censoring threshold of 1.0 mm compared to 0.4 mm resulted in either increases or decreases in similarity to the group mean for different subjects, with little correlation to mean motion (Figure 9F). Similar results were obtained when the similarity was computed using the Euclidean distance between each subject's functional connectivity matrix and the group mean rather than the Pearson's correlation (see Supplementary material).

Similar results were obtained with and without global signal regression (GSR). The similarity to the group mean functional connectivity was slightly higher with GSR, with a mean similarity (Pearson's correlation) of 0.54 with GSR compared to 0.53 without GSR (*p* < 1e-12) (see Supplementary material).

## Discussion

Several datasets were identified by the quality control procedures as having deviations from expected parameters or other issues. Whether a subject should be excluded or not from further group analyses depends on the particular issue, whether this issue can be addressed, and the goals of the study. For example, excluding subjects with abnormal brain anatomy (e.g., enlarged ventricles) may be advisable in studies attempting to characterize typical functional connectivity, but not in studies where such deviations are more common or of interest.

Data that had a different spatial resolution from others at that site can still be processed since all of the data are aligned and re-interpolated to a common resolution in template space, and the current study is already combining data from multiple sites which had acquired data at different spatial resolution. The data from site 500 with the higher spatial resolution (matrix size of 128 voxels vs. 80 voxels) did have greater smoothness, but the impact of this is reduced by smoothing all of the data to a similar final smoothness.

The echo-planar images from 2 subjects were flipped in the I-S direction. This may have resulted from either an error in the conversion of the DICOM files to NIFTI format, or in erroneously setting the subject position in the scanner as supine-feet-first rather than supine-head-first. This flip can in principle be easily corrected, but the process is a bit more complex since the data were acquired with an oblique orientation. In addition, one needs to check whether the left-right orientation is also flipped. This could be done by comparing the alignment of the original and flipped versions of the EPI to the T1. Flips in the left-right orientation were identified in 2 additional subjects. It is unclear whether the error is in the EPI or the T1, but may be determined by examining the original DICOM files. These four subjects were designated as “uncertain”—if the correct left-right orientation can be determined, then they can be included; if the correct orientation cannot be determined then they should be excluded.

A motion censoring threshold of 0.2 mm is commonly used in the field. However, the findings here suggest that this threshold is too stringent for the current study, likely due to the reduced degrees of freedom with aggressive censoring. The similarity of each subject's functional connectivity to the group mean is increased using a threshold of 0.4 mm and this similarity is no longer correlated with the mean motion, which was the case for the more stringent thresholding of 0.2 mm. Relaxing the threshold to 0.4 mm did not increase the correlation of the functional connectivity with motion (QC-FC). Similarly, there was no observable distance dependence of QC-FC at all three motion censoring thresholds evaluated.

Bandpass filtering between 0.01 and 0.1 Hz (or in some studies 0.008–0.08 Hz) is commonly performed in the field. The rationale for this processing step is that the fluctuations of interest typically occur at very low temporal frequencies (<0.1 Hz) (Biswal et al., 1995; Cordes et al., 2001), while non-neuronal fluctuations such as cardiac and respiratory fluctuations occur at much higher frequencies. However, with the typical acquisition rates (repetition times, TR), this physiological noise is aliased to lower frequencies and is not necessarily reduced by the bandpass filtering. Furthermore, bandpass filtering significantly reduces the degrees of freedom, which can affect the quality of the functional connectivity estimates (e.g., see Figure 11). The similarity of the functional connectivity to the group mean increases for nearly all subjects when no bandpass filtering is performed (this is the case regardless of which group connectivity matrix is used for comparison—with vs. without bandpass filtering). When stringent (0.2 mm) motion censoring is applied, the similarity to the group mean is much greater without bandpass filtering compared to with bandpass filtering, particularly in higher motion subjects. This is likely due to the very low degrees of freedom in high motion subjects with both a stringent motion censoring threshold and bandpass filtering. The degrees of freedom are higher without bandpass filtering, which is likely the reason for an increase in similarity (compared to with bandpass filtering) in the higher motion subjects. At a more relaxed (0.4 mm) motion censoring threshold, the similarity does not depend on the mean motion, but is increased (by varying amounts) for nearly all subjects.

The similarity of a subject's functional connectivity to the group mean is a useful way to identify outliers and to determine appropriate processing steps and quality control criteria (e.g., bandpass filtering, motion censoring threshold). A useful qualitative QC step is to visualize the functional connectivity maps from key seed regions (e.g., seed regions from the posterior cingulate to identify the default mode network) and see if they match the expected patterns. While not performed in the current study, quantitative metrics could be computed to measure how well the patterns of these seed-based connectivity maps match the expected pattern. An extension of this approach, in order to measure connectivity for multiple regions throughout the brain is to compute a connectivity matrix from a systematic brain-wide parcellation of the brain and examine the similarity of each subject's connectivity matrix to the group mean connectivity matrix. However, it is important to keep in mind that the goal in many functional connectivity studies is to determine the association of individual differences in functional connectivity with some other variable. That is, we want individual differences in functional connectivity, but not those that are due to differences in subject motion. For that reason, we used the correlation of the similarity with subject motion as a guide to determine the appropriate QC criteria (motion censoring threshold), rather than using the similarity as a QC cutoff. In addition, this measure of similarity may not capture all artifacts, such as systematic errors across the entire sample.

The benefits from a motion threshold of 0.4 mm compared to 0.2 mm found here does not necessarily generalize to all other studies, in particular those acquiring much larger number of time points. The low similarity observed in many subjects in this study is due to the very low degrees of freedom remaining when more aggressive censoring is applied, particularly in combination with bandpass filtering. In studies like HCP and ABCD, where the TR is lower and many more time points have been acquired, there may be sufficient degrees of freedom left for robust estimation of functional connectivity even with more aggressive motion censoring.

The inclusion of global signal regression resulted in a statistically significant, although small, increase in the similarity of each subject's functional connectivity matrix to the group mean. This could reflect improved denoising from GSR. However, because of the lack of ground truth in resting-state functional connectivity, one should be cautious about using only QC criteria to guide processing choices. If any of the nuisance regressors (global signal, CSF signal, or white matter signal) contain effects of interest then regressing them could distort functional connectivity estimates despite improving QC metrics.

Another commonly used QC criteria is to exclude participants with large or “gross” motion, that is, if any frame-to-frame displacement exceeds a predefined threshold, such as 0.55 mm (Satterthwaite et al., 2012, 2013) or 5 mm (Parkes et al., 2018). The motivation behind this exclusion criterion is that larger motion is more likely to be associated with B0-field changes, spin-history effects, and RF coil sensitivity effects. However, if such large motion occurs relatively infrequently (e.g., only a few times during an imaging run), a recent study has shown ways to reduce the effects of this large motion (Birn et al., 2022). For this reason, the maximum motion was not used as a strict exclusion criterion in the current QC study, but simply to flag potential subjects whose functional connectivity maps should be more closely examined for potential artifacts.

Another common problem is the alignment of the EPI data to template space. Since the alignment of the T1 weighted structural images in template space was highly similar across subjects, the errors in the EPI alignment likely result from challenges in aligning the EPI to the T1. Errors in the EPI-to-template alignment were easy to identify using qualitative measures (visualization of the data), but we were not able to find any quantitative metrics that could accurately capture these errors. Misalignments between the EPI and T1 could potentially be reduced by adjusting the EPI-to-T1 alignment cost function or adjusting the parameters of the brain extraction. For example, removal of non-brain tissue (“brain extraction” or “skull-stripping”) that is too aggressive can cause clipped regions of the T1 to be stretched to fit the boundaries and gyri of the template brain. This is not often as visible on the aligned T1s (since the borders of the brain match), but can cause EPI data that is well-aligned to the T1 to be pushed outside the template brain. The subject identified as having a misalignment was designated as “uncertain” since modified processing may result in a better alignment. Whether this subject should be excluded or included depends on the effort an investigator is willing to expend to find the processing options that result in an accurate alignment.

While the current study did not include B0-field maps, studies that do include such measures could use both qualitative and quantitative QC metrics to look at the effectiveness of B0-field distortion correction. For example, the EPI and T1 could be compared before and after correction to verify that the distortion correction was applied in the correct orientation (as determined by the phase encoding direction and polarity) and by the correct amount (as determined by the echo spacing). A Dice coefficient between the EPI and T1 could quantify this QC measure.

Qualitative measures, such as visualizing the data at different points during the processing pipeline, are an indispensable tool for quality control. One reason for this is the myriad number of ways that the processing can go awry. This quality control step can be quite time consuming, and therefore the challenge, particularly for large studies, is making this process as efficient as possible. One way to do this is to concatenate one image (e.g., T1, EPI, or connectivity map in template space) from each subject, and then scroll through the subjects manually or in a movie format. This procedure was quite useful in identifying subjects where the alignment of the EPI to template space was not ideal. These errors in alignment were not captured very well by the Dice coefficient between the EPI and T1-weighted image. This may be because the Dice coefficient between the EPI and T1 is also reduced by B0-field associated signal dropout in the orbitofrontal and temporal lobes, which vary across subjects depending on the shape of the subject's head, the angle of the head to the direction of the magnetic field, and the obliquity of the slice prescription. This signal dropout results in a lower Dice coefficient even with an accurate alignment between the EPI and T1-weighted image.

Many of the measures discussed above are provided with the QC output from the AFNI tool *afni_proc.py*. This QC output includes an alternative way to visualize the alignment of the EPI-to-T1 and T1-to-template—as outlines of the sulci and gray/white matter boundaries on top of either the EPI or the aligned T1. Since *afni_proc.py* was designed to output QC from individual subject data, it does not provide a movie of the alignment across subjects. However, such a movie could easily be generated by extracting one volume (of the EPI, T1, or connectivity map in template space) from each subject and concatenating the datasets. Alternatively, the image snapshots provided by afni_proc's QC could be concatenated into a movie. Such movies can be particularly useful in identifying outliers in the alignment in a large group of subjects.

## Conclusions

A number of quality control procedures and criteria are recommended for the analysis of resting-state functional MRI data. First, it is important to visualize the data at multiple points in the processing pipeline. The accuracy of alignment to template space can be evaluated by concatenating one brain volume from each subject, and then scrolling through the subjects manually or in a movie format. Similarly, outliers in functional connectivity can be determined by concatenating functional connectivity maps from key seed regions in the brain that are known to be part of robust functional networks consistently observed across different subjects—specifically the posterior cingulate to identify the default mode network, primary motor cortex to identify the motor network, primary visual cortex to identify the visual network, and primary auditory cortex to identify the auditory network. Useful quantitative measures include the temporal signal-to-noise ratio, the degrees of freedom remaining after motion censoring and nuisance regression, and the total duration data remaining after motion censoring. While band-pass filtering of the data is currently the standard in the field, future studies may want to re-evaluate the use of this processing step particularly in studies that acquire limited amount of data. Finally, the quality control thresholds used should be examined for each study and may need to be adjusted based on the total amount of acquired data. For example, the QC cutoff of 4 min of good data and 15 degrees of freedom was based on the duration of the runs that were part of the study. Ideally one would want as much data as possible for the best reliability, but this needs to be balanced with the amount of data available and the amount of denoising desired. It is essentially a trade-off between including in the group analysis fewer subjects with “cleaner” data (fewer artifacts) or more subjects with (potentially) noisier data. The balance of this trade-off depends on the levels of motion and other artifacts and the success of noise reduction approaches.

## Data availability statement

The raw data supporting the conclusions of this article are available at https://osf.io/qaesm/ (DOI 10.17605/OSF.IO/QAESM).

## Author contributions

RB conceived of the ideas, performed the analyses, and wrote the manuscript.
