# Supplementary material for: Quality control procedures and metrics for resting-state functional MRI
Source: Front Neuroimaging. 2023 Mar 13;2:1072927. doi: 10.3389/fnimg.2023.1072927 (PMC10406233; doi:10.3389/fnimg.2023.1072927)
Supplement: Supplementary file 1 [file Data_Sheet_1.pdf]

Supplementary Materials

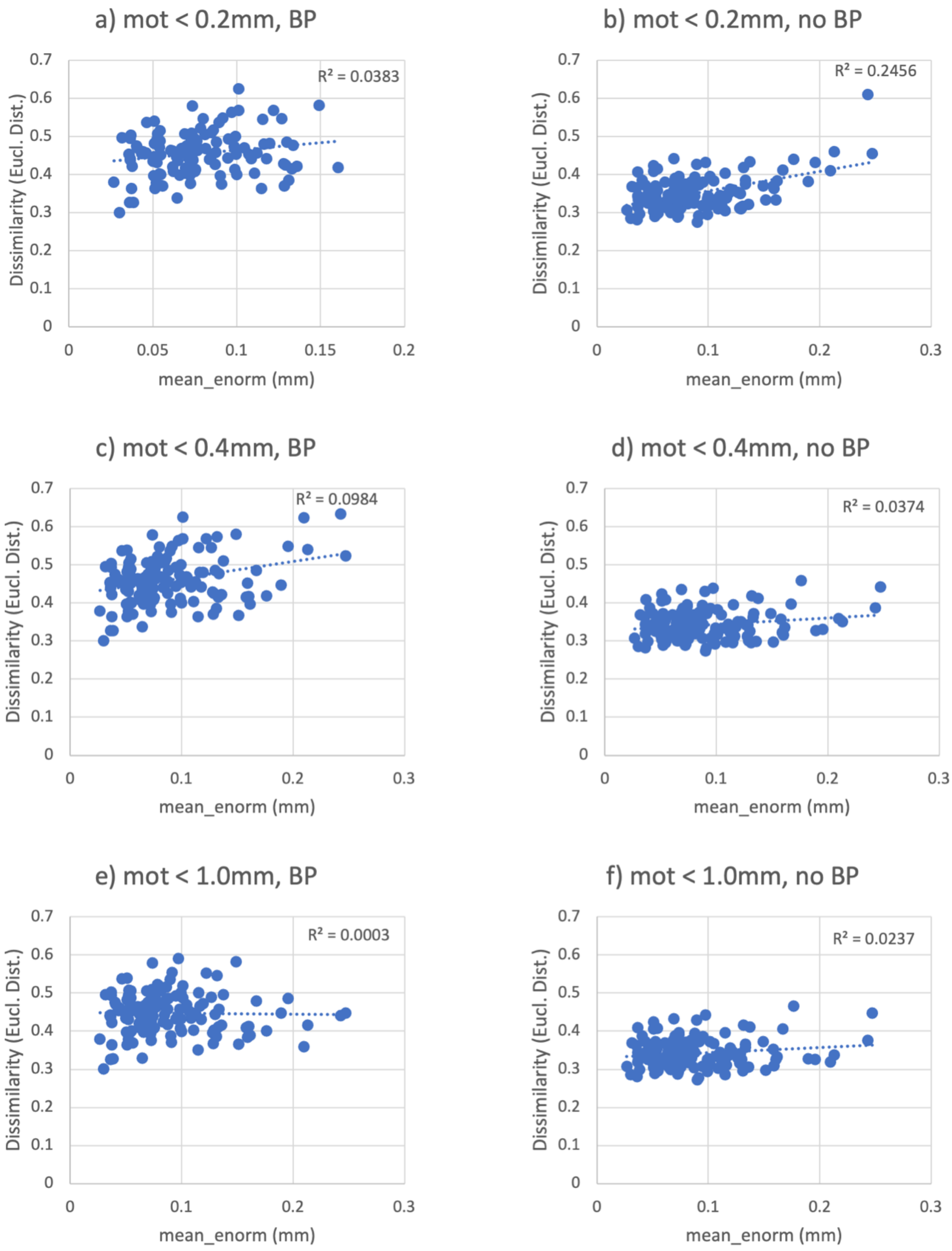

Figure S1: The dissimilarity (Euclidean distance) between each subject’s functional connectivity matrix and the group-average functional connectivity matrix for different motion censoring

thresholds (0.2mm, 0.4mm, 1.0mm) with and without bandpass filtering (BP). BP = bandpass filtering (0.01-0.1Hz), no BP = no bandpass filtering. a) at a motion censor threshold of 0.2mm with bandpass filtering, subjects with higher motion (mean Enorm) show greater dissimilarity. b) without bandpass filtering, dissimilarity is decreased (similarity is increased), but subjects with higher motion still show greater dissimilarity. c) at a motion censoring threshold of 0.4mm with bandpass filtering, dissimilarity to the group mean connectivity again shows a slight positive association. d) Without bandpass filtering, there is again a weak correlation with motion. e) at a motion censoring threshold of 1.0mm and bandpass filtering, there is very little correlation between the similarity and motion. f) Without bandpass filtering at a motion threshold of 1.0mm, there only a slight correlation with motion across subjects.

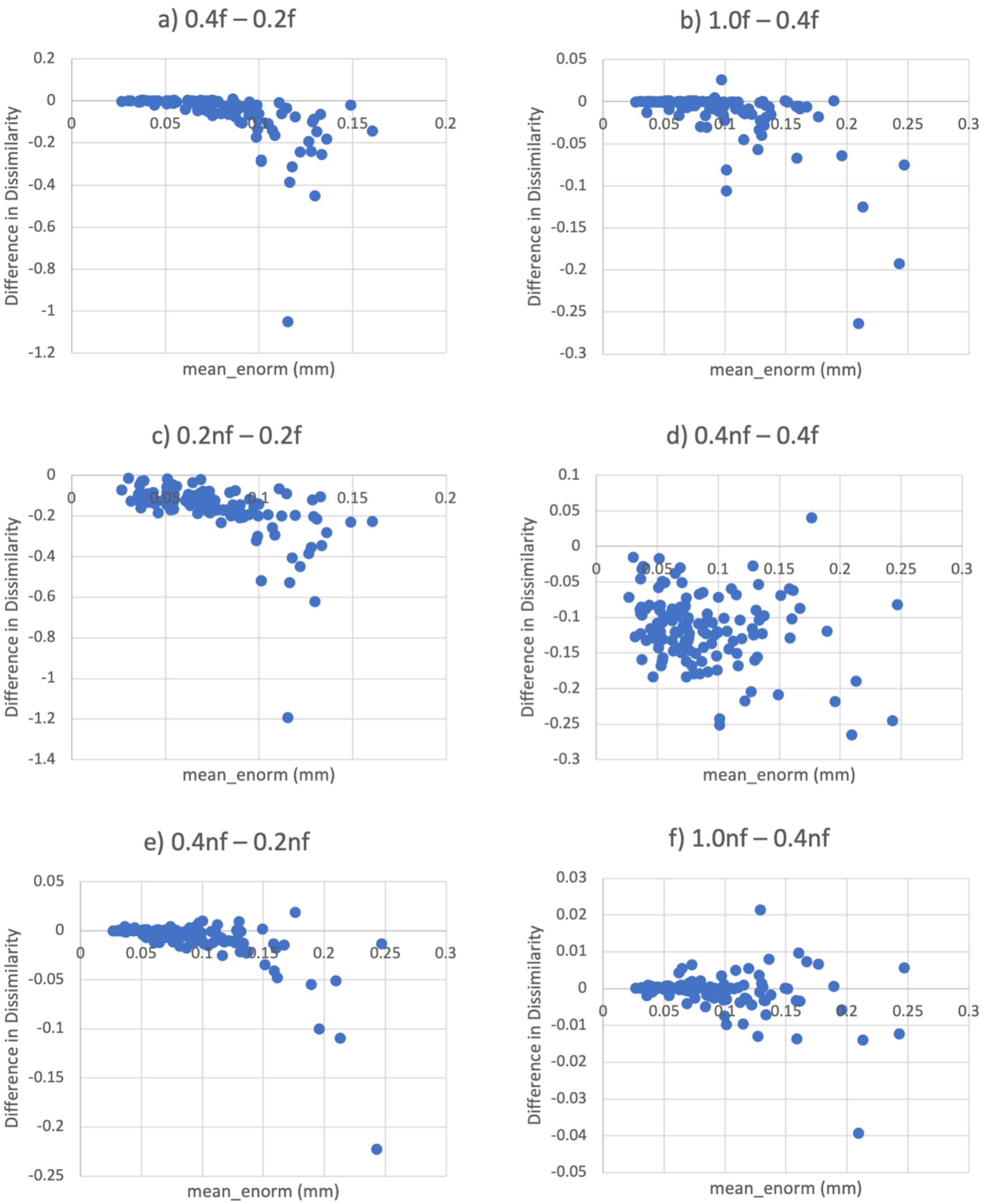

Figure S2: Difference in the dissimilarity (Euclidean distance) of each subject's functional connectivity matrix to the group mean for different levels of motion censoring, with (f) and without (nf) bandpass filtering. **a)** With bandpass filtering, dissimilarity is decreased (similarity is increased) for connectivity matrices computed at a motion threshold of 0.4mm versus 0.2mm, particularly in subjects with high motion. **b)** Similarly with bandpass filtering, dissimilarity is decreased (similarity is increased) for a motion censoring threshold of 1.0mm compared to

0.4mm, particularly for high-motion subjects. **c)** at a motion-censoring threshold of 0.2mm, not performing bandpass filtering decreases dissimilarity (increases the similarity) compared to performing bandpass filtering, particularly in high-motion subjects. **d)** at a motion-censoring threshold of 0.4mm,dissimilarity to the group-mean is decreased (similarity is increased) for most subjects without vs. with bandpass filtering, but less dependent on the mean level of motion. **e)** without bandpass filtering, a motion censoring threshold of 0.4mm has lower dissimilarity (greater similarity) than a threshold of 0.2mm, particularly for high-motion subjects. **f)** Without bandpass filtering, using a motion censoring threshold of 1.0mm compared to 0.4mm can result in either increases or decreases in similarity to the group mean, with little correlation to mean motion.

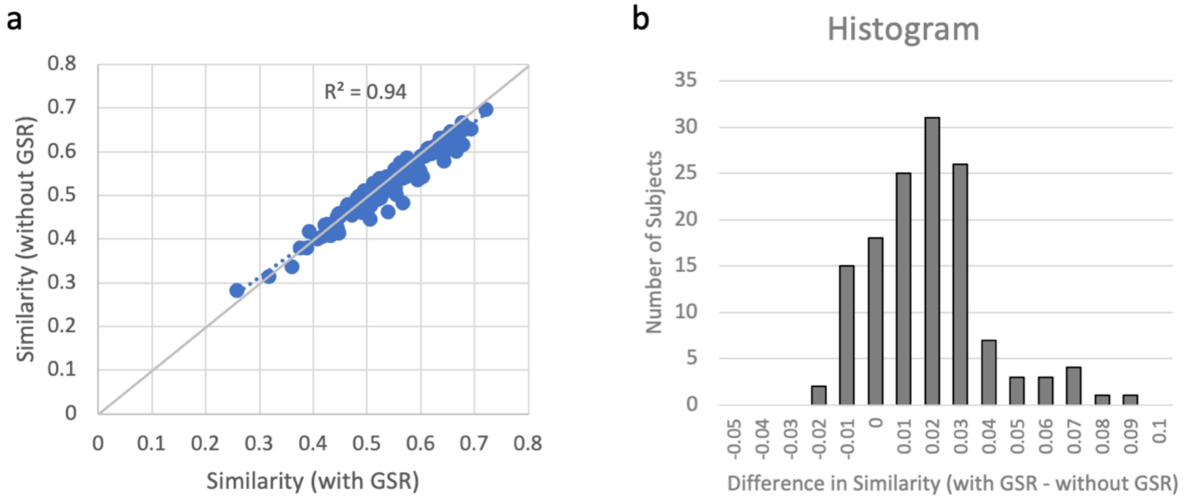

Figure S3: a) Similarity of each subject's functional connectivity matrix to the group mean functional connectivity matrix (Pearson's correlation) with vs without global signal regression (GSR). The two measures are highly correlated (R2=0.94). b) Histogram of the difference in similarity to the group mean with vs without GSR. Most subjects show greater similarity to the group mean connectivity matrix with GSR compared to without GSR.
